# Effects of Lower Limb Constraint Induced Movement Therapy in People With Stroke: A Systematic Review and Meta-Analysis

**DOI:** 10.3389/fneur.2021.638904

**Published:** 2021-03-23

**Authors:** Auwal Abdullahi, Steven Truijen, Naima A. Umar, Ushotanefe Useh, Victor A. Egwuonwu, Tamaya Van Criekinge, Wim Saeys

**Affiliations:** ^1^Neurological Rehabilitation Unit, Department of Physiotherapy, Bayero University Kano, Kano, Nigeria; ^2^Department of Rehabilitation Sciences and Physiotherapy, University of Antwerp, Wilrijk, Belgium; ^3^Department of Physiotherapy, Muhammad Abdullahi Wase Teaching Hospital, Kano, Nigeria; ^4^Lifestyle Diseases Research Entity, Faculty of Health Sciences, North-West University, Mmabatho, South Africa; ^5^Department of Medical Rehabilitation, Nnamdi Azikiwe University, Awka, Nigeria

**Keywords:** stroke, lower extremity, constraint induced movement therapy, activities of daily living, quality of life

## Abstract

**Background:** Constraint induced movement therapy (CIMT) is effective at improving upper limb outcomes after stroke.

**Aim:** The aim of this study was to carry out a systematic review and meta-analysis of the effects of lower limb CIMT studies of any design in people with stroke.

**Materials/ Method:** PubMED, PEDro, OTSeeker, CENTRAL, and Web of Science were searched from their earliest dates to February 2021. Lower limbs CIMT studies that measured outcomes at baseline and post-intervention were selected. Sample size, mean, and standard deviation on the outcomes of interest and the protocols of both the experimental and control groups were extracted. McMaster Critical Review Form was used to assess the methodological quality of the studies.

**Result:** Sixteen studies with different designs were included in this review. The result showed that lower limb CIMT improves functional, physiological and person's reported outcomes including motor function, balance, mobility, gait speed, oxygen uptake, exertion before and after commencement of activities, knee extensor spasticity, weight bearing, lower limb kinematics and quality of life in people with stroke post intervention. However, there were only significant differences in quality of life in favor of CIMT post-intervention [mean difference (MD) = 16.20, 95% CI = 3.30–29.10, *p* = 0.01]; and at follow-up [mean difference (MD) = 14.10, 95% CI = 2.07–26.13, *p* = 0.02] between CIMT and the control group. Even for the quality of life, there was significant heterogeneity in the studies post intervention (*I*^2^ = 84%, *p* = 0.01).

**Conclusion:** Lower limb CIMT improves motor function, balance, functional mobility, gait speed, oxygen uptake, weigh bearing, lower limb kinematics, and quality of life. However, it is only superior to the control at improving quality of life after stroke based on the current literature.

## Introduction

Constraint Induced Movement Therapy (CIMT) is a translational motor rehabilitation technique following injury of the Central Nervous System (CNS). The technique originated many decades ago from use in primates; and was translated to humans following stroke and other neurological conditions (1). The original concept involved constraint of the unaffected limb and forced use of the affected one (2). Subsequent studies in humans involved voluntary massed tasks or shaping practices with the affected limb. Consequently, CIMT has been reported to be effective at improving real world arm use, motor function, and kinematic outcomes by inducing changes in the functions and structures of the brain (3–7). However, there have been many modifications over the years of the original protocol of CIMT, including but not limited to the length of time for the tasks practice, the constraint, and the use of a transfer package (7–9).

The effects of CIMT on the recovery of motor function of the upper limb have been well-investigated (7, 10). The practicability of the protocol for upper limbs could be because of the unilateral nature of the use of these limbs in most of our activities of daily living (ADL). For the lower limbs, this may seem difficult since humans are bipedal, and this requires them to use the two limbs simultaneously for ADL especially during walking. However, the positive results in the recovery of motor function of the upper limb following CIMT persuaded the neuroscientific community to consider translating the technique to the lower limbs. Consequently, a lower limb CIMT protocol was designed to comprise mainly of intensive practice with the affected limb, shaping activities, transfer package, and encouraging the increased use of the affected limb (11). So far, there are several small sample size studies that have investigated the effects of lower limb CIMT on gait parameters, balance, and motor function using different study designs such as case reports, experimental studies, quasi-experimental studies, and randomized controlled trials (RCTs) (12). These studies reported that lower limbs CIMT improved gait speed, step length, motor function, functional mobility, balance, and kinematic outcomes. However, small sample size studies may overestimate the effect of an intervention (13–15). Second, the only difference in the protocols of the CIMT and control groups was the use of a constraint in the CIMT group, with no difference in the types of tasks used in most of these studies, including the intensity. According to Abdullahi, task practice is the most important component of CIMT (16, 17). Therefore, it is possible that the effects of lower limb CIMT reported in those studies were overestimated.

In addition, in upper limb CIMT constraint is used to immobilize the unaffected limb to prevent movement at joints essential for the functioning of the limb. This is to done to maximize the use of the affected limb, and to help recover function. However, for lower limb CIMT, the types of constraints used include encouraging weight bearing on the affected limb, the use of an insole in the affected limb, the use of knee braces or a splint, and attaching weight to the ankle of the affected limb (12). Constraining one of the limbs may cause asymmetry which could negatively affect normal functions such as walking, especially since humans are bipedal. The aim of this study was to therefore carry out a systematic review and meta-analysis on the effects of lower limb CIMT on outcomes after stroke such as gait parameters, balance, motor function, functional mobility, and quality of life. This review sought to answer this question: What are the effects of lower limb CIMT on this information is important as, to date, there does not seem to be any review and/ or meta-analysis on the effects of lower limb CIMT following stroke.

## Methods

The systematic review and meta-analysis were registered with PROSPERO (CRD42017083886).

### Eligibility Criteria and Information Sources

A systematic literature search was carried out in PubMED, PEDro, OT Seeker, CENTRAL, and Web of Science from their earliest dates to February 2021. Similarly, the reference lists of the included studies and a review article were also manually searched for relevant studies. The search terms used were; constraint induced movement therapy, constraint induced therapy, forced use, stroke and lower limbs. The search terms were combined using appropriate Boolean operators such as AND and/or OR where appropriate. The search was also limited to studies published in English only, and those that were carried out in humans. The search was carried out by AA and TVC independently; TVC also removed duplicate studies using Endnote software. The search strategy is available in Appendix 1. Studies of any type of design that included stroke patients who were ≥18 years of age with motor impairment of the lower limbs, and assessed outcomes such as motor function, walking speed, and balance were included in the review. For RCT designs, the studies were included if they compared CIMT with any control interventions. Details of the inclusion and exclusion criteria for the eligibility of the studies are summarized in Table 1.

**Table 1:** Inclusion and exclusion criteria.

**Categories**	**Inclusion criteria**	**Exclusion criteria**
Population characteristics	(1) Stroke patients with motor impairment of the lower limbs, (2) Patients who are ≥18 years, and (3) Sample size ≥1	(1) Patients with bilateral hemiplegia, and (2) Patients with lower limb deformity prior to stroke
Study design	Any type of design in which outcomes were measured before and after intervention	
Measurement variables	Behavioral outcomes measures related to the recovery of lower limbs function such as balance, motor function, functional mobility, and kinematics.	
Interventions	Constraint induced movement therapy, forced use and any interventions that used constraint of the affected limb to aid with the use of the paretic limb	
Language	Studies in english language	

### Selection of Eligible Studies and Extraction of Data

The study selection was carried out by AA and NAU independently using Rayyan software (18). At first, the abstracts and titles of the studies were assessed, and in the absence of sufficient information to either include or exclude a study, full texts of the articles were read. Disputes on whether to include or exclude studies were resolved through consensus discussions between AA and NUM or through consulting another author (VAE). Data extraction was carried out by AA and the data included were study designs, sample size, stage of stroke, participants' mean age, interventions for both experimental and control groups, including intensity and duration, and outcomes assessed (mean scores and standard deviation).

### Assessment of the Methodological Quality of the Included Studies

Methodological quality of the included studies was assessed using the Modified McMaster Critical Review Form for Quantitative Studies (19, 20). This form is used to assess: (1) whether the purpose of the study was clearly stated, (2) whether the relevant literature was reviewed, (3) the extent to which the sample of the study was described, (4) whether the sample size in the study was justified, (5) randomization, (6) whether the procedure for the randomization was appropriate, (7) how reliable the method used to establish diagnosis of the condition is, (8) how valid the outcome measures used are, (9) how reliable the outcome measures used are, (10) whether the intervention used was described in detail, (11) avoidance of contamination, (12) whether co-intervention was avoided, (13) whether statistical significance was reported, (14) whether the method of analysis used was appropriate, (15) whether clinical significance or importance was reported, (16) whether drop-outs were reported, and (17) whether the conclusion was drawn appropriately in accordance with the study methods and results. The scores for each item ranges from zero to one. A score of zero is awarded when the answer to the question is no or is not addressed; whereas a score of one is awarded when the answer to the question is yes. However, when a question is not applicable to a particular design such as studies that are not RCTs, the answer is indicated as not applicable (NA). In addition, the Cochrane risk of bias table was used to further assess the risk of bias of the included RCTs, and the results of this are presented in a risk of bias graph. The assessment was carried out by two of the authors (AA & NAU) and any disputes were resolved through discussions and contacting a third reviewer (VAE). The level of evidence of the included studies was determined using the National Health and Medical Research Council's (NHMRC) evidence hierarchy (21).

### Results Synthesis and Meta-Analysis

Preferred Reporting Items for Systematic Reviews and Meta-Analyses (PRISMA) was used to report the results of this systematic review and meta-analysis. RevMan (version 5.3) was used to create the PRISMA flow chart of the study and the graph for the risk of bias of the included RCTs. In addition, the mean and standard deviation of the scores on the outcomes of interest post intervention and at follow up; and the study sample size (for both the experimental and the control groups) were pooled using RevMan (version 5.3). When studies used the same outcome measures, the data was analyzed using fixed effect model. However, when studies used different outcome measures, the data was analyzed using the random effect model. Heterogeneity between studies was considered substantial only when *I*^2^ (which measures whether the percentage of variation across studies is caused by heterogeneity rather than chance) value is ≥50%. Furthermore, a level of significance, *p* < 0.05 was considered to be significant. For interpretation of the findings and their implication for clinical practice, the NHMRC form methodology was used (21).

## Result

### Study Selection

**Figure 1:**
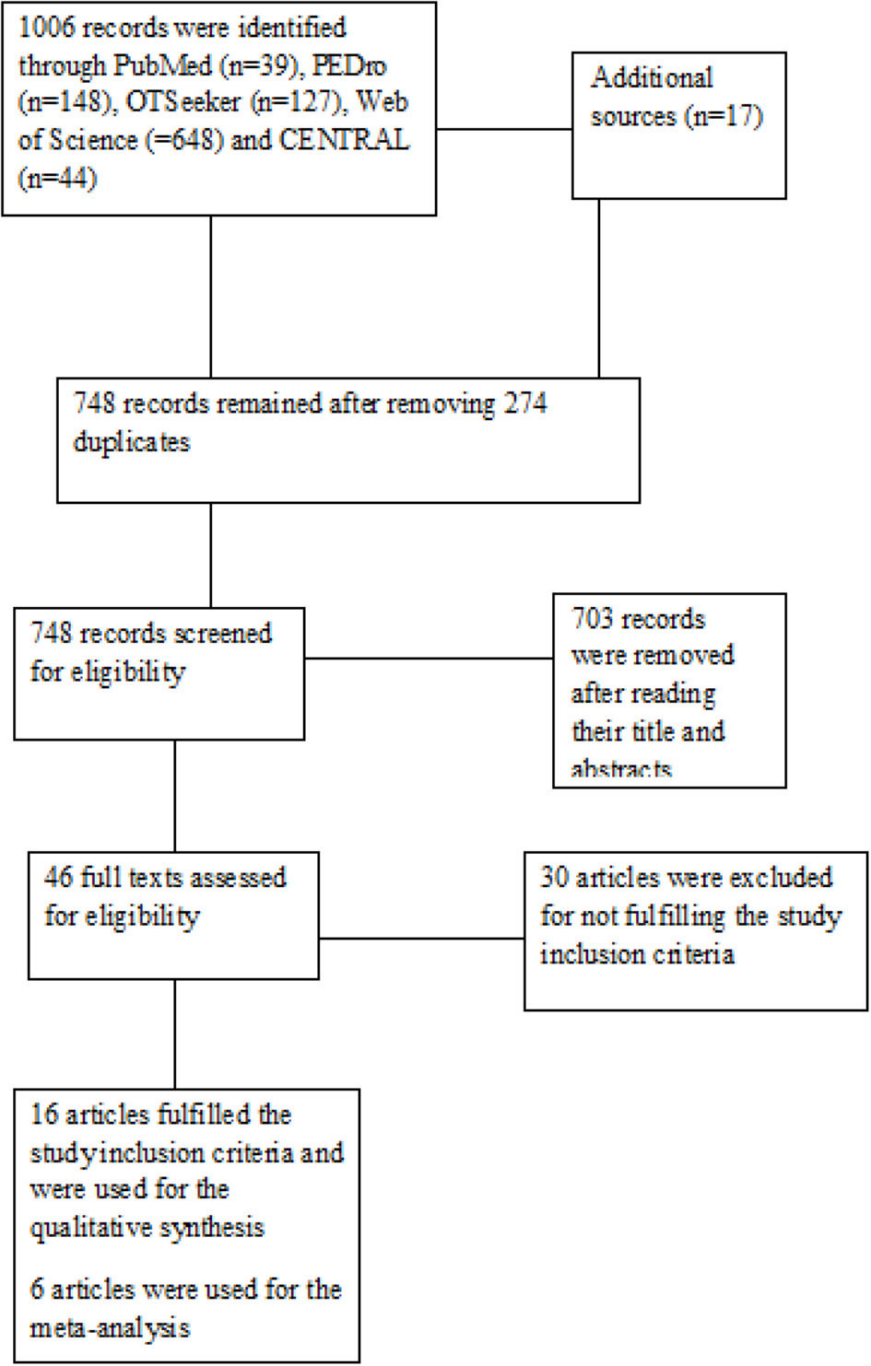
The Study flowchart.

A total of 16 studies were included in the study (22–37). The search of the databases and the reference list of the relevant studies yielded 1,023 hits in which 17 hits were provided from the reference list of the relevant studies. Subsequently, full texts of 46 articles were read and 30 articles were excluded for not fulfilling the study inclusion criteria. See Figure 1 for the study flowchart.

### Characteristics of the Included Studies

The total number of participants in the included studies was 304, out of which 169 and 135 were men and women, respectively. The range for the sample size in the studies was between one and 58 participants. However, only four studies reported how the sample size was calculated (22–24, 37). The studies were published between 2005 and 2021. Out of the 16 included studies, 10 are RCTs (22, 24–31, 37), and two studies each are either single subject experimental AB designs (32, 33); pre-test- post-test experimental designs (23, 34); and case reports (35, 36), respectively.

Eight studies included chronic stroke patients (23, 24, 26, 31–34, 36). Three studies included subacute stroke patients (25, 29, 35). Two studies included subacute and chronic stroke patients (22, 28). Three studies included acute, subacute, and chronic stroke patients (27, 30, 37). Overall, the range of time since the stroke was 1 week to 6 years. Eleven out of the 16 studies included participants who could independently walk several meters or more (23, 25–28, 30, 32–34, 36, 37). One study included participants who could rise from a chair without using the arm or stand independently (24). Four studies included participants who were within Brunnstrom stages of recovery, 3 to 4 (22, 28, 29, 36).

In addition, 12 studies included participants with no significant cognitive impairment (22, 24, 26–32, 34, 36, 37). One study included participants who had significant walking speed deficits of ≤0.8 m/s (30). Only 11 studies provided information on the types of stroke the participants suffered, 176 ischaemic and 44 haemorrhagic (22, 23, 25, 27, 28, 30, 31, 34–37). Eight studies used participants with ischaemic and haemorrhagic stroke (22, 23, 27, 28, 30, 31, 34, 37). Two studies used participants with only ischaemic stroke (25, 35). This information is important since the type of stroke may provide information on the prognosis (38). Furthermore, most of the studies excluded participants who had serious medical conditions such as unstable hypertension, musculoskeletal deformities, and cardiopulmonary conditions that could hinder participation in the studies.

The studies used different forms of task practice and constraints in the intervention. For the constraint, one study used a weight attached to the participants' ankles (30). Four studies emphasized that participants bear weight on the affected limb during training (23, 24, 27, 34). Two studies used shoe insoles (26, 28). Three studies used knee braces or a splint (25, 31, 35). Two studies used whole leg orthosis (32, 33). One study used whole leg orthosis and a shoe insert (22). One study asked participants not to make use of the unaffected during training as much as possible (37). However, in one study, no constraint was used (36). For the affected side, only one study with 38 participants did not provide information on the affected side (30). Therefore, excluding this study, 143 and 123 participants had right and left sided hemiplegia, respectively. The affected side is an important prognostic indicator after stroke (39). In addition, some of the outcome measures used in the studies include electromyography for muscle activity (motor function), lower limb Fugl-Meyer for motor function, lower extremity motor activity log (LE-MAL) for real world use of the lower limb, Berg balance scale (BBS) for balance, the stroke specific quality of life questionnaire (SSQOL), and the stroke impact scale (SIS) for quality of life, the 10 m walk test (10MWT) for walking speed, the 6 min walk test (6MWT) for walking endurance, the motion analysis system for gait analysis, the timed up and go test (TUG) and Rivermead mobility index (RMI) for functional mobility, and the hard activity chart for exertion. Motor function is the ability to have voluntary control of movement patterns (40). Functional mobility ‘is a person's physiological ability to move independently and safely in a variety of environments to accomplish functional activities or tasks and to participate in the activities of daily living, at home, work and in the community’ (41). See Table 2 for the details of the characteristics of the included studies.

**Table 2:** Characteristics of the studies.

**References**	**Design**	***N***	**Stroke phase**	**Mean age (years)**	**Intervention**	**Outcomes**	**Findings**
Gatti et al. (25)	RCT	10	Subacute	55.5 ± 12.9	Standing from and sitting on a chair, performing mini-squats, and maintaining a standing posture during DIMT and control phases for 6 h per day. A shoe wedge was also worn on the affected limb to offset asymmetry	Spatiotemporal parameters (stride length, stride speed and swing phase asymmetry index). sEMG of tibialis anterior, medial gastrocnemius, rectus femoris, vastus medialis, gluteus medius, and biceps femoris	There was significant improvement in spatiotemporal parameters post intervention Better improvement in the number of correct activations during DIMT
Jung et al. (27)	RCT	21	Acute, subacute, and chronic	CIMT = 56.4 ± 11.1 Control = 56.3 ± 17.1	Gait training, 30 min per day, 5 times a week for 4 weeks. The experimental group used a cane that provided auditory feedback to enhance weight bearing on the affected limb	Muscle activities of gluteus medius and vastus medialis (sEMG). Gait speed and single-limb support phase (Electronic Walkway System)	Significant increase in muscular activity and gait speed in the experimental group. Significant decrease in weight bearing on the cane and improvement in single limb support phase in the experimental group
Aruin et al. (26)	RCT	18	Chronic	57.7 ± 11.9	Muscle strengthening exercises, sit to stand and stand to sit, weight shift on the affected side, stepping forward, sideways, backward on a stool and walking ones in a week, 60 min per session in the experimental and control groups for 6 weeks. Experimental group wore a full-shoe 0.6 cm insole on the unaffected side	Weight bearing (NeuroCom Balance Master), balance (BBS), motor recovery (FMT), gait velocity (10MWT)	Significant improvement in weight bearing, balance and gait velocity in the experimental group. No significant difference in motor recovery between groups
eSilva et al. (30)	RCT	38	Acute	27 to 70 years	Load discharge exercises in anterior posterior and latero-lateral directions, 3 sets of 15 repetitions and 30 min of treadmill training per day for 9 days in both experimental and control groups. The non-paretic limb was constrained with a mass equivalent of 5% body weight in the experimental group	Balance (BBS), functional mobility (TUG), spatiotemporal, and kinematic parameters (Qualisys motion systems)	All outcomes improved in both groups. However, there was no difference between groups in all outcomes
Yu et al. (28)	RCT	21	Subacute and chronic	FUT = 56.8 ± 11.0 CPT = 54.2 ± 11.1	CPT received gait correction, treadmill training, postural training, and other training activities for functional mobility FUT received custom-fitted wedged insole to raise the lateral border of the unaffected foot to 5°. Circuit training- sit to stand, stepping over blocks in different directions, walking on inclined treadmill, climbing stairs, and walking over various surfaces with obstacles. The exercises in both groups were carried out for 90 min per day, 5 times a week for 2 weeks	Gait performance and mobility (PWV, FWV, SSI, TSI, TUG, and RMI). Quality of life (SSQOLTV). Walking velocity was measured and gait parameters were derived using an electronic walkway system	FUT provided greater improvement in most gait parameters. However, there was no significant difference in quality of life and TSI
Numata et al. (35)	Case report	1	Subacute	72 years	mCIMT consisting of balance, walking and weight bearing exercises for 40 min per day. Constraint with knee splint for 13 h per day	Patient's self-report and observation	Improved use of the affected limb
Choi et al. (24)	RCT	36	Chronic	GB CIMT = 61.25 ± 5.59 GB = 62.58 ± 5.51 Control = 61.92 ± 6.08	GM CIMT and GB groups received did Ski slalom and soccer heading for 30 min a day, 3 times a week for 4 weeks. In addition, they received traditional therapy for 60 min a day, 5 days a week for 4 weeks. However, the GB CIMT group constrained the unaffected limb by reducing weight bearing on the limb	Weight bearing symmetry (WBBs) and MatLab program, balance (FRT), limits of lateral stability (mFRT), and functional mobility and dynamic balance (TUG)	GBT CIMT produced better effects on static balance, weight bearing symmetry, and side to side weight shift
Danlami and Abdullahi (31)	RCT	18	Chronic	sCIMT = 48.2 ± 7.89 tCIMT = 55.67 ± 9.00 Control = 54.14 ± 6.87	sCIMT performed 480 repetitions of functional tasks per day. tCIMT performed the same functional tasks for 2 h per day. Control group received usual physiotherapy for 2 h per day. Interventions in each group were carried out 5 times a week for 4 weeks	Lower limb motor impairment assessed using lower limbs Fugl Meyer	sCIMT demonstrated higher improvement in motor impairment
Zhu et al. (29)	RCT	22	Subacute	mCIMT = 59.18 ± 7.35 Control = 58.00 ± 6.97	Both control and mCIMT groups received standard care 5 times a week for 4 weeks. The mCIMT received gait training for 2 h per day in addition	Gait parameters measured using 16 Camera Eagle Motion Analysis System	mCIMT improved gait parameters and center of mass displacement in both sagittal and frontal planes
Kallio et al. (33)	Single subject experimental AB design	3	Chronic	71 to 76 years	Phase consisted of baseline period of 2 weeks; balance, motor function, functional mobility, and walking ability were measured 3 times each. Phase B consisted of 2 h of mCIMT, 5 times a week for 4 weeks. Outcomes measurement took place twice each week	Dynamic balance was measured using step test. Motor function was measured using Fugl Meyer. Functional mobility (TUG). Walking ability (6MWT)	mCIMT may improve balance and motor function
Vearrier et al. (34)	Pre-test- post-test	10	Chronic	59.0 ± 18.0	6 h per day intensive massed tasks practice for 10 consecutive days	Center of pressure (COP) and time to stabilization (TTS) of the COP measured using AMLAB data acquisition system. Balance (BBS) and (Activities specific balance confidence scale)	Decreased TTS post intervention and prolonged reactive balance
Marklund and Klassbo (32)	Single subject experimental AB design	5	Chronic	62.0 ± 13.69	Phase consisted of baseline period of 2 weeks. Outcomes were measured 3 times each week. Phase B consisted of 6 h of functional training per day for 2 weeks. Outcomes were measured 3 times each week and at 3 and 6 months follow up	Balance, motor function, functional mobility, and weight bearing asymmetry were assessed using step test, Fugl Meyer, TUG, and weighing scale, respectively	Intensive massed practice improved balance, motor function. mobility, weight bearing asymmetry, and walking ability
Billinger et al. (23)	Single pre-test- post-test AB design	12	Chronic	60.6 ± 14.5	Isokinetic flexion/extension protocol using Biodex System (Single Leg Exercise) for 40 repetitions per set with 30 s rest breaks in between each set. Participants were instructed to self-progress with the goal of reaching 40 sets. Exercise was carried out 3 times a week for 4 week	Cardiopulmonary fitness, gait velocity, motor function, lean tissue mass, and knee extensor strength were measured using maximal exercise test, 10-meter fast walk test, Fugl Meyer, DEXA, and Biodex system	Oxygen uptake (VO_2_) and gait velocity improved post-intervention
Acaroz Candan and Livanelioglu (22)	RCT	30	Subacute and chronic	CIMT = 55.13 ± 14.70 Control = 57.67 ± 12.20	mCIMT and NDT for experimental and control groups, respectively, 1.5 h, 5 times a week for 4 weeks. For the experimental group, the unaffected limb was immobilized with whole leg orthosis and 1cm shoe raise for 90% of the waking hours	Muscle strength (Motricity index), Quality of life (SSQoL and SIS), amount of perceived recovery (VAS)	All the outcomes improved better in the CIMT group
dos Anjos et al. (36)	Case report	1	Chronic	56	Intensive training of the affected limb, shaping practice, and transfer package adopted from upper limb CIMT, 3.5 h per day for 10 consecutive days. No constraint was used	Real world use of the lower limb (LE-MAL), balance (BBS), walking endurance (6MWT), and walking speed (10MWT)	Positive changes in all outcomes that seemed to attain minimal clinically important difference
Abdullahi et al. (37)	RCT	58	Acute, subacute, and chronic	Group 1 = 50.2 ± 13.9 Group 2 = 47.8 ± 14.7	repCIMT = 600 repetitions of tasks practice per day. hCIMT = tasks practice for 3 hours per day. Both groups carried out the tasks 5 times weekly for 4 weeks. The tasks carried out in both groups were: stepping forward, backward stepping, side stepping, ball kicking, and stair climbing	Motor impairment (LE-FM), balance (BBS), functional mobility (RMI), knee extensor spasticity (MAS), walking speed (10MWT), and endurance (6MWT) and exertion before and after commencement of activities (hard activity chart)	All the outcomes improved post-intervention in both groups. However, the repCIMT group had better improvement in knee extensor spasticity and exertion before and after commencement of activities

*DIMT, Disadvantaged limb induced movement therapy; sEMG, Surface Electromyography; BBS, Berg balance scale; FMT, Fugl Meyer test; 10MWT, Ten meter walk test; TUG, Timed up and go test; TUG, Timed up and go test; SSQOLTV, Stroke specific quality of life, Turkish version; RMI, Rivermead mobility index; PWV, Preferred walking velocity; FWV, Fast walking velocity; TSI, Temporal symmetry index; SSI, Spatial symmetry index; FUT, forced use training; CPT, Conventional physical therapy; GB, Game based; sCIMT, Standardized CIMT; tCIMT, Traditional CIMT; WBB, Wii balance board; FRT, Functional reach test; mFRT, Modified functional reach test; TUG, Timed up and go test; TUG, Timed up and go test; DEXA, Dual energy X-ray Absorptiometry; NDT, Neurodevelopmental therapy; SSQoL, Stroke specific quality of life questionnaire; SIS, Stroke impact scale; VAS, Visual analog scale; LE-MAL, Lower extremity motor activity log; BBS, Berg balance scale; 6MWT, Six minute walk test; 10MWT, 10 minutes walk test; LE-FM, lower limb Fugl Meyer; BBS, Berg balance scale; RMI, Rivermead mobility index; MAS, Modified Ashworth scale; 10MWT, ten meter walk test; 6MWT, six minute walk test*.

### Quantitative Synthesis

A total of six RCTs were included in the meta-analysis (22, 24, 26, 28, 30). One study has one experimental and two control groups (24). Four RCTs were excluded from the meta-analysis (25, 29, 31, 37). Two of the RCTs were excluded because they did not provide sufficient information to enable a meta-analysis (25, 29); while the remaining two were excluded because they compared two different modes of CIMT with one as a control to the other (31, 37).

**Figure 2:**
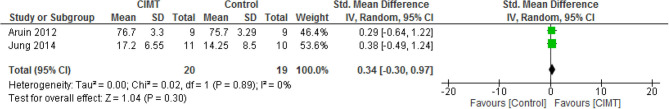
Motor function post-intervention.

For motor function, there was no significant difference between CIMT and the control [standardized mean difference (SMD) = 0.34, 95% CI = −0.30–0.97, *p* = 0.30]. In addition, there was no significant heterogeneity in the studies (*I*^2^ = 0%, *p* = 0.89). See Figure 2 for the forest plot.

**Figure 3:**
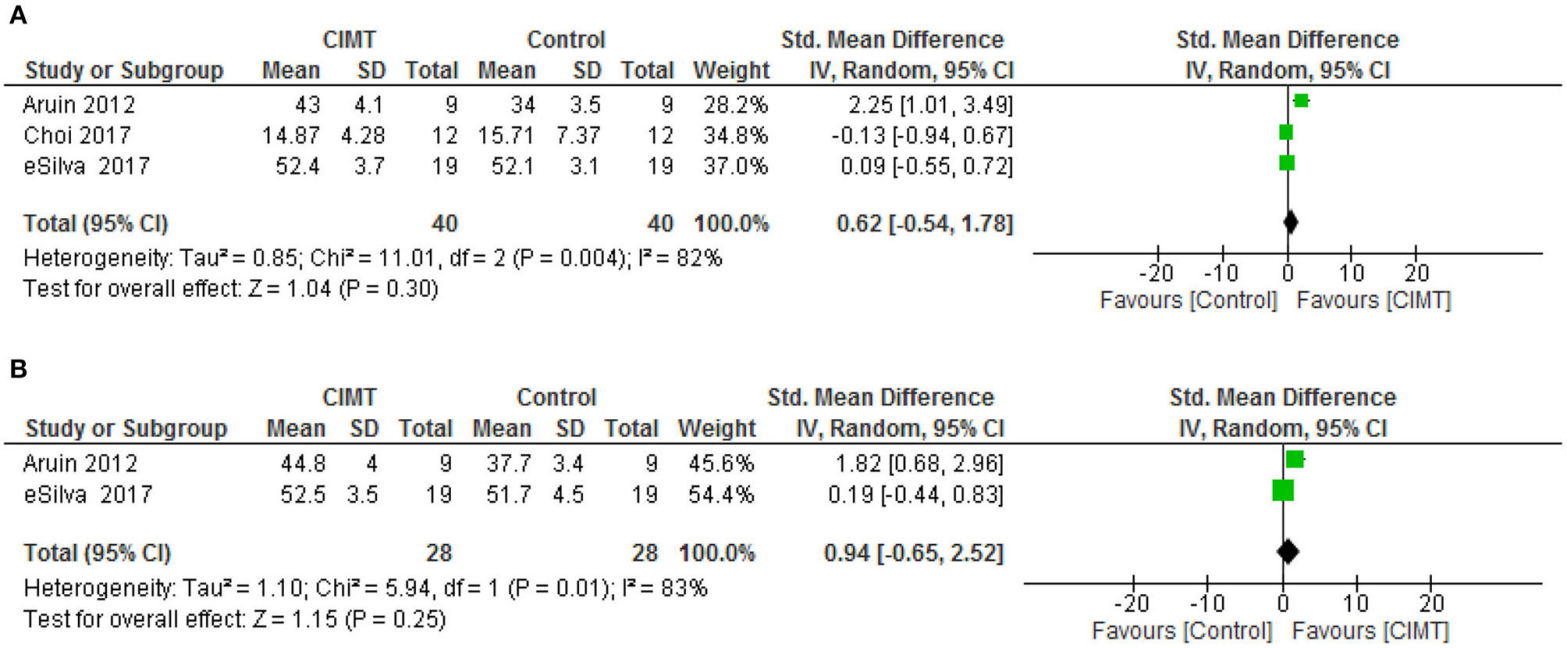
**(A)** Balance post-intervention. **(B)** Balance at follow up.

For balance post intervention and at follow-up, there was no significant difference between CIMT and the control (SMD = 0.62, 95% CI = −0.54–1.78, *p* = 0.30) and (SMD = 0.94, 95% CI = −0.65–2.52, *p* = 0.25), respectively. However, there was a significant heterogeneity in the studies, (*I*^2^ = 82%, *p* = 0.004) and (*I*^2^ = 83%, *p* = 0.001), respectively. See Figures 3A,B for the forest plots of balance post intervention and at follow up, respectively.

**Figure 4:**
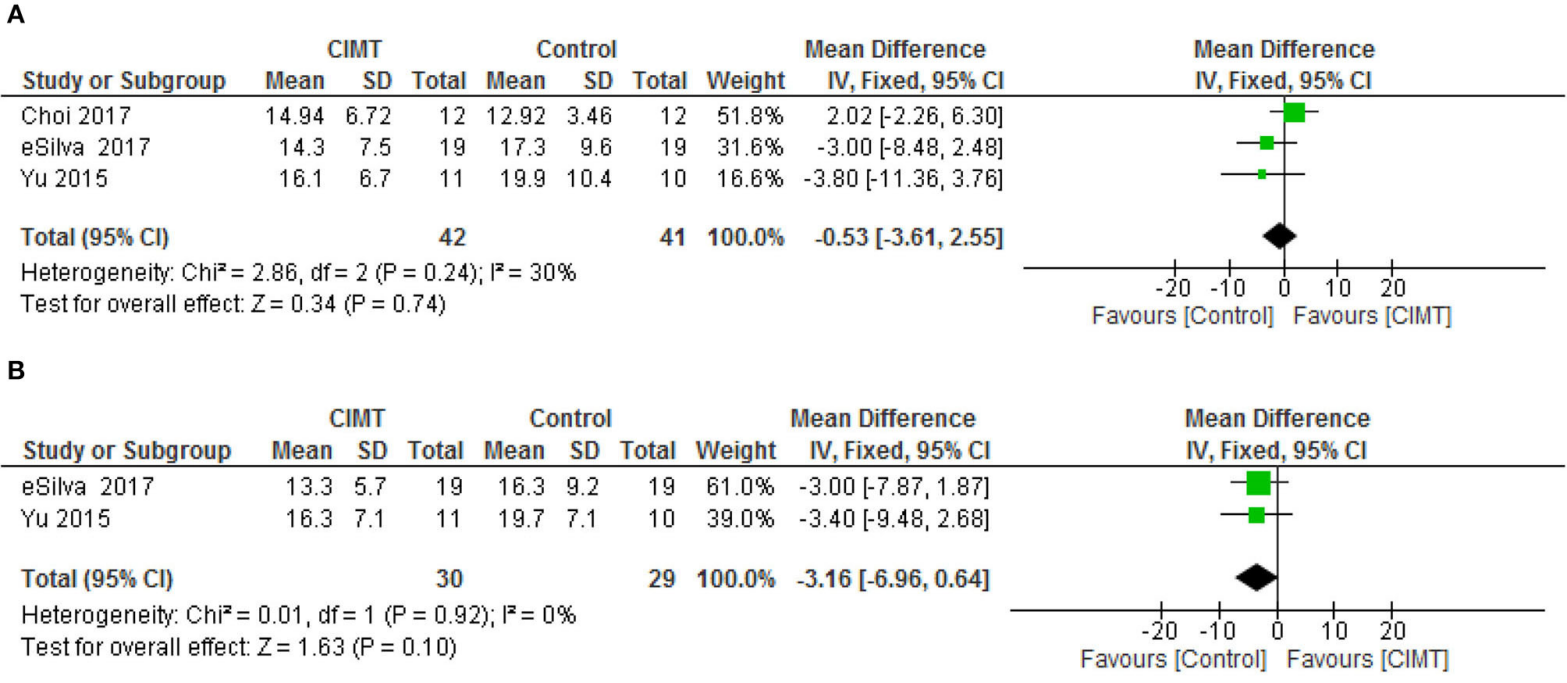
**(A)** Functional mobility post-intervention. **(B)** Functional mobility follow up.

For functional mobility, there was no significant difference between CIMT and the control at both post intervention [mean difference (MD) = −0.53, 95% CI = −3.61–2.52, *p* = 0.74] and follow-up [MD = −3.16, 95% CI = −6.96–0.64, *p* = 0.10). However, there was no significant heterogeneity in the studies, (*I*^2^ = 30%, *p* = 0.24) and (*I*^2^ = 0%, *p* = 0.92), respectively. See Figures 4A,B for the forest plots of functional mobility post intervention and at follow up, respectively.

**Figure 5:**
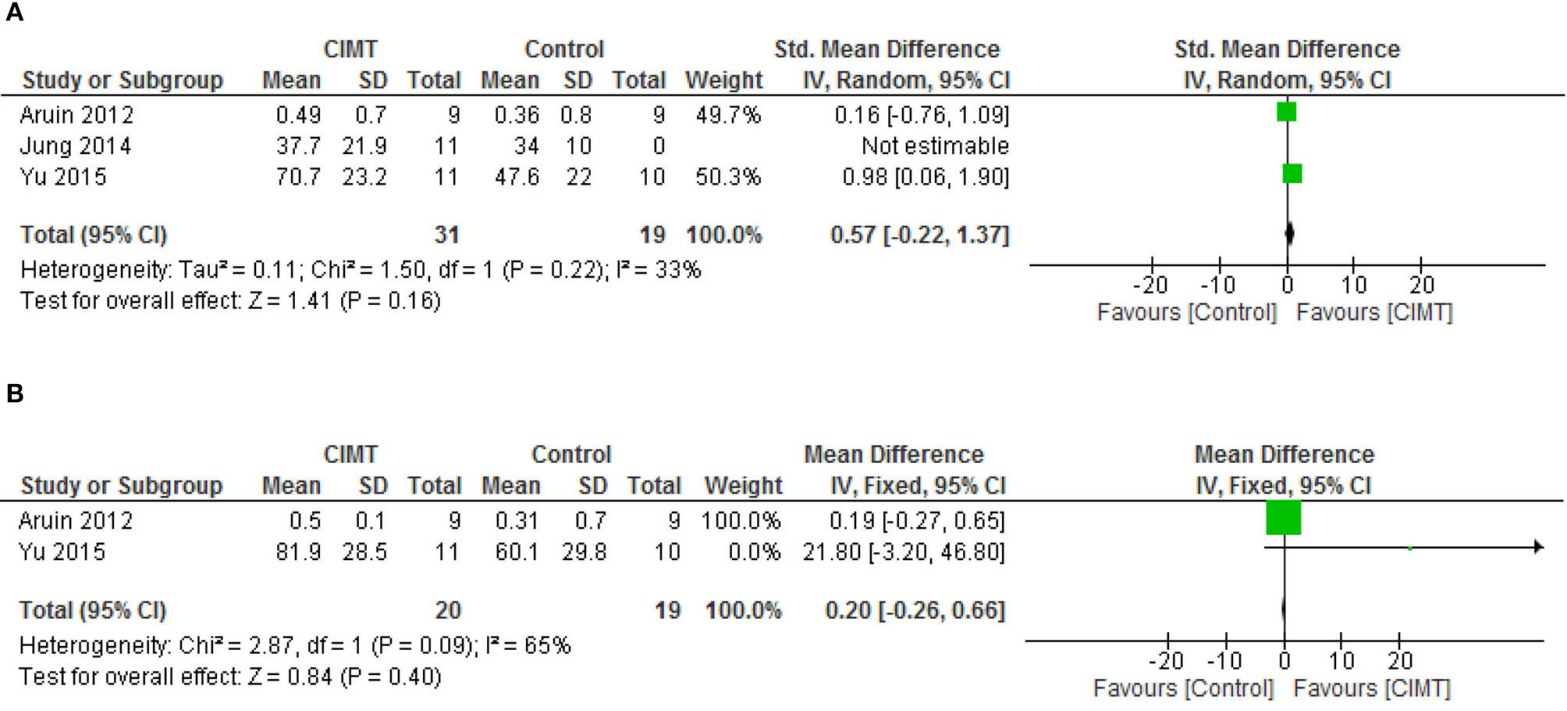
**(A)** Gait speed post-intervention. **(B)** Gait speed at follow up.

For gait speed, there was no significant difference between CIMT and the control post intervention (SMD = 0.57, 95% CI= −0.22–1.37, *p* = 0.16) and at follow-up (SMD = 0.20, 95% CI = −0.26–0.66, *p* = 0.09). However, there was no significant heterogeneity in the studies, (*I*^2^ = 33%, *p* = 0.22) and (*I*^2^ = 65%, *p* = 0.09), respectively. See Figures 5A,B for the forest plots of gait speed post intervention and at follow up, respectively.

**Figure 6:**
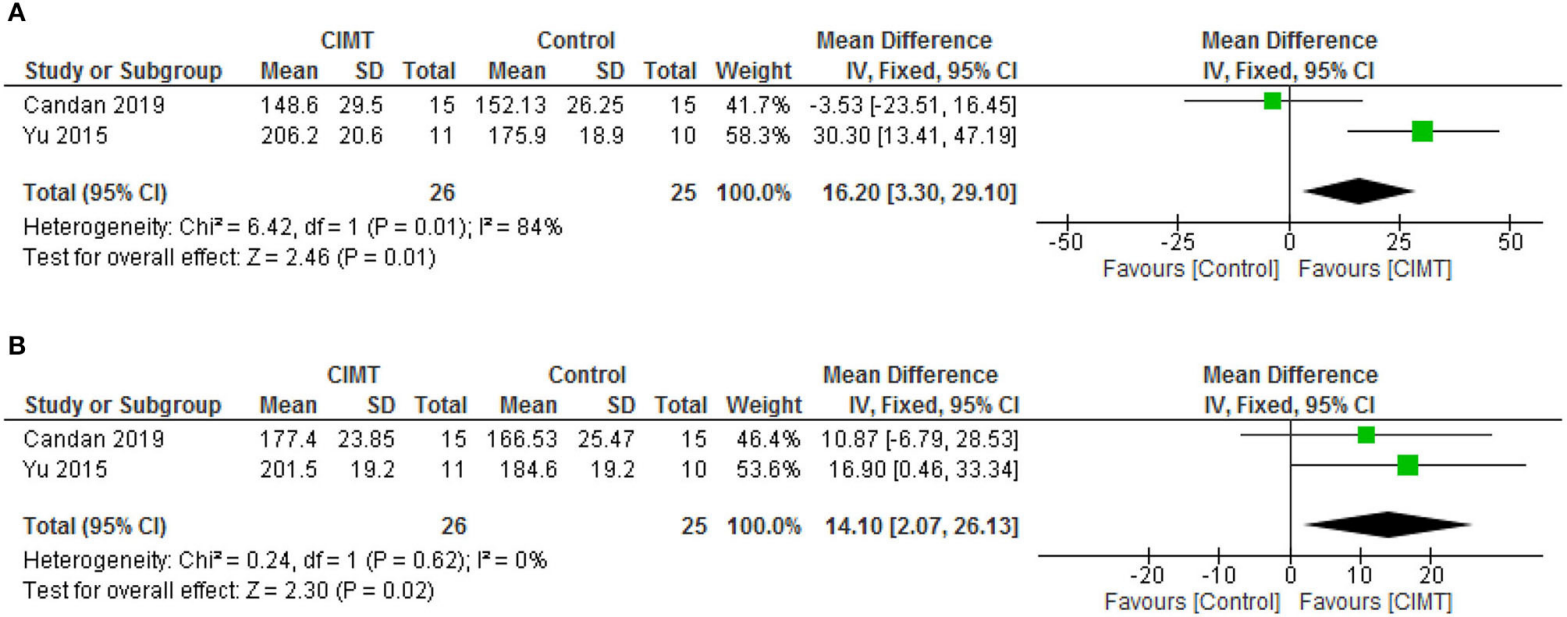
**(A)** Quality of life post-intervention. **(B)** Quality of life at follow up.

For quality of life, there was significant difference between CIMT and the control post intervention (MD = 16.20, 95% CI = 3.30–29.10, *p* = 0.01) and at follow-up (MD = 14.10, 95% CI = 2.07–26.13, *p* = 0.02) in favor of CIMT. However, there was significant heterogeneity in the studies post intervention (*I*^2^ = 84%, *p* = 0.01). See Figures 6A,B for the forest plots of quality-of-life post intervention and at follow up, respectively.

### Level of Evidence and Risks of Bias of the Included Studies

**Figure 7:**
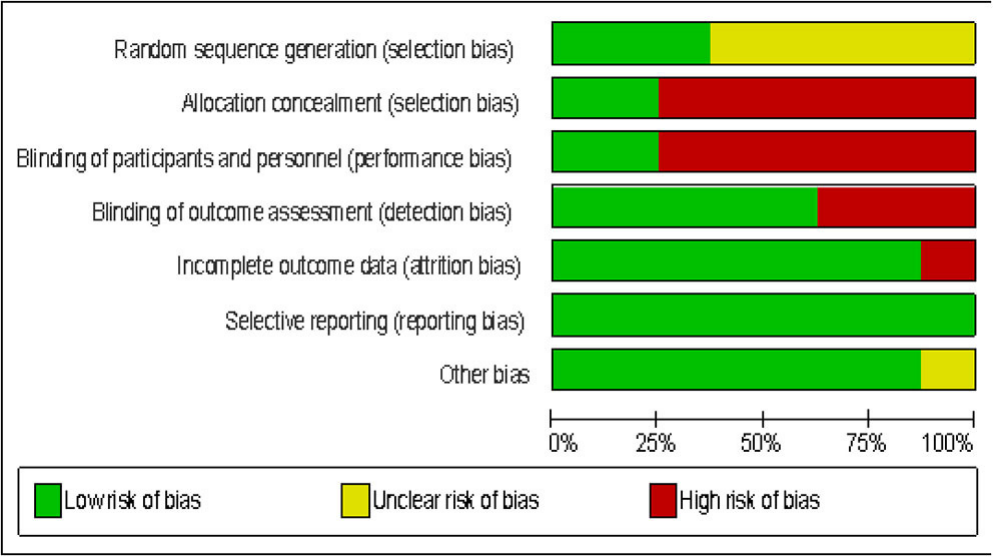
Risk of bias of the included RCTs.

Ten studies were rated as Level II evidence (22, 24–31, 37). One study was rated as Level III-2 evidence (34). Three studies were rated as level III-3 evidence (23, 32, 33). Two studies were rated as level IV evidence (35, 36). The main methodological concerns in the included studies are a lack of justification for the sample size as only four studies performed a power calculation (22, 24, 27, 37); only six studies provided information on the reliability (24–27, 34, 36); only four studies provided information on validity (26, 34, 36, 37); and lack of reporting clinical significance as only three studies provided information on this (22, 23, 36). In addition, only three studies reported adverse events, tiredness, and stiff and aching muscles; physical and mental stress; and mild low back pain and calf muscle pain, respectively (33, 35, 37). See Table 3 for the details of the methodological quality and level of evidence of the included studies and Figure 7 for the risk of bias graph of the included RCTs. Considering the results of the review, the evidence was interpreted in Table 4 using NHMRC form (42).

**Table 3:** Levels of evidence and methodological quality of the included studies.

**References**	**Design**	**Level of Evidence**	**1**	**2**	**3**	**4**	**5**	**6**	**7**	**8**	**9**	**10**	**11**	**12**	**13**	**14**	**15**	**16**	**17**	**Total score**
Gatti et al. (25)	RCT	II	Yes	Yes	Yes	No	Yes	Yes	Yes	Yes	No	Yes	No	Yes	Yes	Yes	No	No	Yes	12/17
Jung et al. (27)	RCT	II	Yes	Yes	Yes	Yes	Yes	Yes	Yes	Yes	No	Yes	Yes	Yes	Yes	Yes	No	No	Yes	13/17
Aruin et al. (26)	RCT	II	Yes	Yes	Yes	No	Yes	Yes	Yes	Yes	Yes	Yes	Yes	Yes	Yes	Yes	No	No	Yes	15/17
eSilva et al. (30)	RCT	II	Yes	Yes	Yes	No	Yes	Yes	Yes	Yes	Yes	Yes	Yes	No	Yes	Yes	No	Yes	Yes	14/17
Yu et al. (28)	RCT	II	Yes	Yes	Yes	No	Yes	Yes	Yes	No	No	Yes	Yes	Yes	Yes	Yes	No	No	Yes	12/17
Numata et al. (35)	Case report	IV	Yes	Yes	Yes	No	NA	NA	No	No	No	Yes	NA	Yes	No	Yes	No	NA	Yes	7/13
Choi et al. (24)	RCT	II	Yes	Yes	Yes	Yes	Yes	Yes	Yes	Yes	No	Yes	Yes	Yes	Yes	Yes	No	No	Yes	14/17
Danlami and Abdullahi (27)	RCT	II	Yes	Yes	Yes	No	Yes	Yes	Yes	No	No	Yes	Yes	Yes	Yes	Yes	No	Yes	Yes	13/17
Zhu et al. (29)	RCT	II	Yes	Yes	Yes	No	Yes	Yes	Yes	No	No	Yes	NA	Yes	Yes	Yes	No	Yes	Yes	13/17
Kallio et al. (33)	Single subject experimental AB design	III-3	Yes	Yes	Yes	No	NA	NA	Yes	Yes	Yes	Yes	No	Yes	No	No	No	No	Yes	9/15
Vearrier et al. (34)	Single subject experimental AB design	III-2	Yes	Yes	Yes	No	NA	NA	Yes	Yes	Yes	Yes	No	Yes	Yes	Yes	No	No	Yes	11/15
Marklund and Klassbo (32)	Single subject experimental AB design	III-3	Yes	Yes	Yes	No	NA	NA	Yes	No	No	Yes	No	Yes	Yes	Yes	No	No	Yes	9/15
Billinger et al. (23)	Within subject design	III-3	Yes	Yes	Yes	No	NA	NA	Yes	No	No	Yes	Yes	Yes	Yes	Yes	Yes	Yes	Yes	12/15
Acaroz Candan and Livanelioglu (22)	RCT	II	Yes	Yes	Yes	Yes	Yes	Yes	Yes	Yes	Yes	Yes	No	No	Yes	Yes	Yes	Yes	Yes	15/17
dos Anjos et al. (36)	Case report	IV	Yes	Yes	NA	NA	NA	NA	Yes	Yes	Yes	Yes	Yes	Yes	No	Yes	Yes	Yes	Yes	12/13
Abdullahi et al. (37)	RCT	II	Yes	Yes	Yes	Yes	Yes	Yes	Yes	Yes	Yes	Yes	Yes	Yes	Yes	Yes	No	Yes	Yes	16/17

**Table 4:** NHMRC form framework.

**Component**	**Grade**	**Comments**
1) Evidence	A-Excellent Several Level II studies	Quantity: a total of 16 studies Participants: 304 stroke patients Level II: 10 studies Level III-2: 1 study Level III-3: 3 studies Level IV: 2 studies
2) Consistency	C-Satisfactory Some inconsistency reflecting genuine uncertainty around clinical question	Consistent reporting of statistical significance [only two studies; (35, 36)] did not report this information) Different study designs, interventions and outcome measures were used which was also indicated by significant heterogeneity (*I^2^* = 50%) in some results of the meta-analyses.
3) Clinical Impact	C-Satisfactory moderate	Thirteen studies reported statistical significance; but only three studies (11, 23, 29) reported clinical significance Two studies (32, 35) reported adverse events
4) Generalizability	B-Good	The population of the studies was similar to the target population (stroke patients)
5) Applicability	B-Good Applicable internationally with just a few caveats	Studies were carried out in 10 countries in 4 different continents
Recommendation	C-Satisfactory (evidence), but it needs to be applied with caution	There is significant heterogeneity between studies

## Discussion

The aim of this systematic review and meta-analysis was to find out what effects of lower limbs CIMT have on outcomes after stroke. The results showed that, 16 studies with levels of evidence ranging from level IV to level II were eligible for the study. The studies were carried out in 10 different countries and four different continents. In addition, the studies showed that, lower limb CIMT improves motor function, balance, functional mobility, walking speed, oxygen uptake, weight bearing, knee extensor spasticity, exertion before and after commencement of activities, quality of life, and kinematic outcomes. However, a meta-analysis involving 6 level II studies showed that there was only a significant difference between the experimental and control groups in quality of life at both post intervention and follow-up, in favor of CIMT. These findings can be explained by many factors. First, the participants in the RCTs included in the meta-analysis were mainly in the chronic stage of stroke, a stage in which the functional recovery process is usually very slow (43). Therefore, during this stage, many patients might have learned to compensate for their deficits, especially since humans are bipedal. To achieve recovery of function during the chronic stage of stroke, motor rehabilitation techniques such as CIMT may need to be combined with sensorimotor stimulation techniques such as transcortical direct stimulation (tCDs) and trans-magnetic stimulation (TMS) (44). This may help in recruiting more areas of the brain that will help in controlling motor function. Coincidently, none of the included studies combined CIMT with any sensorimotor stimulation techniques.

Second, there is heterogeneity in the included studies, especially in the protocols of CIMT and the outcome measures used. Heterogeneity can affect outcomes in terms of either overestimating or underestimating them (45). For instance, types of constraints (shoe raise, weight bearing on the affected limb, splints/orthosis, weight attached to the participants' ankles, and whole leg orthosis) used in the studies differ significantly. Use of constraints such as a shoe raise or insole during lower limb CIMT could alter the lower limbs' biomechanics and hinder recovery of function. Therefore, considering, the potential of the use of constraints such as the shoe raise in altering lower limb biomechanics, it can be argued that use of a constraint during lower limb CIMT may not be necessary. Rather, patients can be instructed to make sure they maximize the use of the affected limb, while minimizing the use of the unaffected limb as much as possible. Similarly, half of the six RCTs included in the meta-analysis are underpowered as only four studies calculated the sample size. Small sample studies may overestimate or underestimate effects (13–15). Furthermore, in most of the studies, there does not seem to be much difference in terms of the types, or the intensity of tasks practiced between the experimental and the control groups. According to the results of previous studies, intensity of task practice (how much a task is practiced or repeated) is an integral requirement for recovery of function (46–48). However, in some of the studies included in this review, intervention was provided for just a short period of time which may not allow for the high intensity training required for neuroplasticity to take place and therefore, the subsequent improvement in functions. Neuroplasticity is usually achieved when there is a high repetition of tasks of about 300 times per day which is usually performed within 1 h on average (46, 47). High repetitions of task practice may only be achieved within a short period when technological aids are used. In a recent systematic review and meta-analysis, task repetitions of 800 to 1,000 were achieved in 30 min in patients with severe motor impairment using exoskeleton robot-assisted gait training (48). Therefore, studies of lower limbs CIMT should be very clear on the types of tasks practiced in both the experimental and the control groups. Equally, the use of the number of repetitions of task practice as the measure of intensity of practice during lower limb CIMT should be encouraged. This is because this type of protocol provides a clear instruction on the intensity of the practice as opposed to the use of number of hours of practice (16, 49). Fortunately, some of the reviewed studies also used this type of protocol (23, 30, 31, 37).

In addition, most of the exercises used in the studies did not target balance directly but were aimed at improving motor function and functional mobility. Balance control goes beyond motor ability (50). However, even though there was no significant difference in motor function and balance between groups, careful observation of the forest plots revealed that the combined effect sizes for these two outcomes were in favor of the experimental group. This indicates that, there was a trend toward better improvement in balance in the experimental group compared with the control group. Similarly, CIMT showed better improvement in quality of life which is an important outcome for people with stroke. The reason for this could be because quality of life is a subjective outcome which may depend on many factors including time since stroke (51). Interestingly, most of the participants in the included studies were within the chronic stage of stroke, a time when patients might have learned to cope with their condition or disability. Consequently, their disabilities may not seriously or negatively impact their quality of life.

Nevertheless, the overall findings have implications for both research and practice. For research, more studies are needed to compare the effects of lower limb CIMT and control intervention on outcomes after stroke. For practice, since the number of repetitions of task practice required for recovery of motor function following stroke is known, lower limbs CIMT should focus on the use of this number of repetitions in their protocols. This type of protocol has been advocated and used for upper limb CIMT with success (16, 17, 46, 47). In addition, use of constraints such as a shoe raise or insole during lower limb CIMT, which could alter the lower limb's biomechanics and subsequently the limb's function, should be discouraged. Furthermore, the neurophysiological underpinnings of lower limb CIMT should also be investigated to help elucidate more robust evidence for it. This is because, for upper limb CIMT, many neurophysiological changes such as an increased cortical map size, decreased intracallosal inhibition, and the upregulation of growth associated protein 43 (GAP-43) have been reported (5, 52, 53).

## Conclusion

Lower limb CIMT is effective at improving outcomes such balance, functional mobility, motor function, gait speed, oxygen uptake, exertion before and after commencement of activities, knee extensor spasticity, weight bearing, lower limb kinematic, and quality of life following a stroke. However, based on the current evidence, it is only superior to the control at improving quality of life. Therefore, more studies, especially RCTs, with adequate power are needed to determine the effects of lower limb CIMT on outcomes after stroke compared with the control. The studies should also include qualitative methodology to help gain more insights from the participants on how lower limb CIMT improves their functions.

## Data Availability Statement

The original contributions presented in the study are included in the article/Supplementary Material, further inquiries can be directed to the corresponding author/s.

## Author Contributions

AA and NU conceived the study. AA, ST, NU, UU, VE, TV, and WS designed the study. AA, NU, TV, and VE in the data collection. AA did the qualitative and quantitative analysis and writing up of the manuscript. ST and WS cross checked the analyses and the interpretations. ST, NU, UU, VE, TV, and WS critically reviewed the manuscript. All authors approved the manuscript for publication.

## Conflict of Interest

The authors declare that the research was conducted in the absence of any commercial or financial relationships that could be construed as a potential conflict of interest.

## Appendix 1

The search strategy used in PubMED and CENTRAL.

1. Constraint induced movement therapy
2. Constraint induced therapy
3. Forced use
4. 1 OR 2 OR 3
5. Stroke
6. 4 AND 5
7. Lower limbs
8. 6 AND 7
